# Gender Differences in Major Risk Factors for Cardiovascular Disease in Mexican Adults

**DOI:** 10.5334/gh.1531

**Published:** 2026-03-16

**Authors:** Lourdes Flores-Luna, Consuelo Escamilla-Núñez, Ivette Cruz-Bautista, Rosalba Rojas-Martínez, Leticia Hernández-Cadena, Lilia Castro-Porras, Martín Romero-Martínez, Carlos A. Aguilar-Salinas

**Affiliations:** 1 Centro de Investigación en Salud Poblacional, Instituto Nacional de Salud Pública, México; 2 Unidad de Investigación en Enfermedades Metabólicas, Instituto Nacional de Ciencias Médicas y Nutrición Salvador Zubirán, México City, México; 3 Centro de Investigación en Políticas, Población y Salud, Facultad de Medicina, Universidad Nacional Autónoma de México, México; 4 Centro de Investigación en Evaluación y Encuestas, Instituto Nacional de Salud Pública, México; 5 Dirección de Investigación, Instituto Nacional de Ciencias Médicas y de Nutrición Salvador Zubirán, México

**Keywords:** cardiovascular, CVD, risk factors, lifestyle, adults, sex differences

## Abstract

**Introduction:**

Unhealthy lifestyle habits, including high-calorie diets and physical inactivity, increase the risk of cardiovascular disease (CVD). Chronic conditions such as hypertension, diabetes, and kidney disease frequently precede cardiovascular events. This study aimed to characterize the sociodemographic and clinical profiles of individuals who have experienced such events and to provide updated evidence on the prevalence of cardiovascular risk factors in the Mexican adult population.

**Methods:**

Data were obtained from adults aged ≥ 20 years with chronic diseases who participated in the 2018 National Health and Nutrition Survey. Biomarkers were measured from fasting (8 h) blood samples. The survey used a cross-sectional, probabilistic design with national representativeness. The prevalence of major cardiovascular risk factors was estimated by sex and CVD diagnosis. Logistic regression analyses yielded adjusted odds ratios, identifying significant associations between key risk factors and CVD.

**Results:**

Women exhibited a greater prevalence of obesity (38.6%), diabetes (17.4%), dyslipidemia (88.5%), and a personal history of myocardial infarction (19.5%) in comparison to men; similarly, women and individuals aged 50–59 years demonstrate the presence of more than two concurrent risk factors. The prevalence of obesity, dyslipidemia and hypertension in women was associated with an increased likelihood of a CVD diagnosis, adjusted odds ratio (aOR) = 2.6[95% CI: 1.5, 4.5].

**Conclusion:**

The prevalence of cardiovascular events was similar between sexes; however, different risk factor profiles were identified. The pharmacological treatment alone has not been sufficient to achieve therapeutic goals.

## Introduction

Cardiovascular disease (CVD) is a leading cause of death and disability worldwide (1), with higher rates among people of low socioeconomic status (2). In 2019, CVD caused 17.9 million deaths, representing 32% of global mortality; 85% were due to heart attacks and strokes (3). In Mexico, 189,210 CVD cases were reported in the first half of 2023, with 53% in men (4). According to data from the National Health Survey (ENSANUT), the prevalence of CVD increased from 2.9% in 2006 to 4.9% in 2022 (5), and in 2023 it was 4.4% among adults aged ≥ 20 years, higher in men (4.9%) and rural areas (4.9%) than in women (4.0%) and urban areas (4.3%) (6).

CVD risk factors are classified as modifiable and non-modifiable. Modifiable factors include excess caloric intake, smoking, and physical inactivity; non-modifiable factors include family history, age, and sex (7). These factors are often related to limitations in access to health care, healthy food, education, and stable employment, all of which are restricted in Mexico’s socially disadvantaged communities, particularly due to pronounced economic and social inequalities (8). The high prevalence of obesity, diabetes, and other cardiovascular risk factors is a consequence of these structural barriers, underscoring the need to address cardiovascular health within a broader socioeconomic framework (9,10). Furthermore, highly prevalent conditions such as obesity, hypercholesterolemia, diabetes, renal impairment, metabolic dysfunction-associated steatotic liver disease, and hypertension substantially contribute to the population burden of CVD (11). There is evidence that cardiovascular mortality rises more rapidly in women during the perimenopausal and menopausal transition (12). During this stage, underdiagnosis and undertreatment are frequent, and lipid targets are less often achieved in women (13). In our country, it remains unknown whether such disparities in cardiovascular risk and its assessment exist.

Several risk factors carry high relative and population-attributable risk for heart failure. Vascular damage from fat accumulation restricts blood flow to organs, causing angina, myocardial infarction, or heart failure (14). In the United States, hypertension, obesity, diabetes, and atherosclerotic CVD affect over 100 million adults, showing the magnitude of the problem (15). Therefore, updated evidence is needed on the prevalence of CVD and its risk factors. This study aimed to quantify their frequency and sex-specific distribution in the Mexican adult population.

## Methods

### Design and study population

Data from adults aged 20 and older with chronic diseases and biomarker results from a blood sample collected after an eight-hour fast were obtained from the National Health and Nutrition Survey 2018 database (Ensanut-2018). The survey employed a cross-sectional design based on probabilistic sampling, ensuring national representativeness. This survey’s methodology has been described elsewhere (16).

The Ethics, Research, and Biosafety Commissions of the National Institute of Public Health approved the methodology of the Ensanut-2018 survey. After the procedures were explained, all participants signed an informed consent form (16).

### Variables definition

The included CVDs were myocardial infarction, angina, and heart failure, and they were identified by self-report through the question: ‘Has a doctor ever told you that you have or have ever had a heart attack, angina pectoris, or heart failure?’

The included socio-demographic characteristics were: sex (men/women), education level (none, elementary school, high school, college, or postgraduate), marital status (single, married or cohabiting, separated, divorced, or widowed), indigenous language speaker (yes/no), health social security, depressive symptomatology (yes/no), and socioeconomic status (low, medium, or high).

For major CVD risk factors, we defined obesity as a body mass index (BMI) ≥ 30 kg/m²; hypertension (HTA) as a systolic blood pressure ≥ 140 mm Hg or diastolic blood pressure ≥ 90 mm Hg or prior clinical diagnosis of arterial hypertension, or current antihypertensive medication use; type 2 diabetes (T2D) according to American Diabetes Association criteria, including a previous diagnosis or fasting glucose ≥ 126 mg/dL, or glycated hemoglobin ≥ 6.5%; dyslipidemia as total cholesterol ≥ 200 mg/dL or LDL-C ≥ 100 mg/dL or HDL-C levels < 40 mg/dL in men, or HDL-C levels < 50 mg/dL in women; family history of heart attack (when father and/or mother had heart attack); and finally, current smoking status (yes/no).

Additionally, risk factors were considered, such as central obesity defined according to the International Diabetes Federation (IDF) criteria for the Mexican population (waist circumference (WC) ≥ 90 cm in men and WC ≥ 80 cm in women); remnant cholesterol levels ≥ 30 mg/dL; physical activity categories (vigorous, moderate, or inactive); excessive drinking patterns (defined as consuming five or more drinks for men or four or more drinks for women in the last 30 days on a single occasion); high risk of CVD according to the Globorisk equation with CKD-risk scores of ≥ 10 points (17); low estimated glomerular filtration rate (eGFR < 60 ml/min/1.73 m^2^); and energy intake measured in kilocalories.

### Statistical analysis

All analyses were presented overall and stratified by sex. To describe the study population, we presented the main characteristics, reporting means or proportions with 95% confidence intervals (95% CI) according to the variable type. Subsequently, we estimated the prevalence of major CVD risk variables. The distribution of the combination of risk factors was shown graphically by sex and age group according to CVD diagnosis, starting with two factors and adding one additional risk factor at a time until all were incorporated. Then, we presented the percentages of therapeutic targets for cardiovascular risk by treatment use.

Finally, to examine the associations between cardiovascular risk factors (obesity, dyslipidemia, hypertension, diabetes, familial history of myocardial infarction, and current smoking status) and CVD (myocardial infarction, angina, and heart failure), we estimated adjusted odds ratios (aORs) with 95% CI using multiple logistic regression (MLR) models. Specific models were built for sex and treatment use. Additional aORs were estimated from MLR models for the combinations of risk factors, starting with obesity and subsequently incorporating an additional risk factor; these models were stratified by sex and treatment use (statins and antihypertensive agents).

All models were adjusted for age, physical activity, total energy intake, and socioeconomic status. For the women model, it also included whether the woman was post-menopausal.

The SVY package in Stata 14.0 was used to consider the intricate sample design in the statistical analysis.

## Results

### Sociodemographic characteristics

A total of 10,503 adults aged 20 years or older were included in the study, representing 82,767 million people with the same characteristics; 45.4% were men with an average age of 43.9 years, and 54.6% were women with an average age of 44.5 years. Regarding education, 60.7% of adults had no education or had completed elementary school, and 6.2% spoke an indigenous language. Regarding security, 12.4% of adults attended a clinic adjacent to a pharmacy, a frequency higher among men (13.4%) than among women (11.5%) (Table 1).

**Table 1:** Sociodemographic characteristics of the adult population by sex. Mexico, Ensanut 2018.


CHARACTERISTIC	ALL	MEN	WOMEN

*Sample size in adults 20 years or more*	10,503	4,502	6,001

*Frequency in thousand*	82,767	37,551	45,216

***Sociodemographic***			

*Age, years (mean)*	44.3 [43.6,44.9]	43.9 [43.1,44.8]	44.5 [43.7,45.4]

*20–39*	43.3 [41.5,45.1]	44.4 [42.0,46.9]	42.3 [39.9,44.7]

*40–59*	36.8 [35.2,38.4]	36.1 [33.9,38.5]	37.4 [35.3,39.6]

*60 and more*	19.9 [18.5,21.4]	19.4 [17.6,21.4]	20.3 [18.5,22.3]

*Education level*			

*None-education*	5.4 [4.8,6.1]	4.4 [3.6,5.3]	6.3 [5.5,7.2]

*Elementary school*	55.3 [53.6,57.0]	53.4 [50.8,55.9]	57.0 [54.7,59.2]

*High school*	21.1 [19.7,22.6]	22.2 [20.0,24.5]	20.2 [18.4,22.3]

*College or higher*	18.1 [16.7,19.6]	20.1 [18.0,22.4]	16.5 [14.8,18.4]

*Marital status*			

*Single*	21.4 [19.9,23.0]	24.3 [22.1,26.7]	18.9 [16.9,21.1]

*Married or living with a partner*	66.7 [65.0,68.3]	68.4 [65.9,70.7]	65.3 [63.0,67.5]

*Separated, divorced, or widowed*	12.0 [11.0,13.0]	7.3 [6.2,8.6]	15.8 [14.4,17.4]

*Indigenous language speaker*	6.2 [5.2,7.4]	6.0 [5.0,7.3]	6.3 [5.1,7.7]

*Usual health insurance*			

*IMSS*	32.9 [31.1,34.7]	33.1 [30.6,35.6]	32.8 [30.6,35.1]

*ISSSTE*	6.0 [5.1,6.9]	5.1 [4.1,6.4]	6.6 [5.5,8.0]

*SSA*	33.8 [32.1,35.5]	31.5 [29.1,33.9]	35.6 [33.4,37.9]

*Pharmacy-Adjacent Clinic*	12.4 [11.1,13.7]	13.4 [11.5,15.7]	11.5 [10.1,13.1]

*Others*	15.0 [13.7,16.3]	16.9 [14.9,19.1]	13.4 [12.0,15.1]

*Depressive symptomatology present*	18.3 [17.1,19.5]	13.8 [12.2,15.6]	22.0 [20.4,23.8]

*Socioeconomic status*			

*Low*	29.1 [27.4,30.9]	29.5 [27.2,32.0]	28.7 [26.7,30.8]

*Medium*	32.7 [31.1,34.4]	32.0 [29.6,34.5]	33.3 [31.2,35.5]

*High*	38.2 [36.2,40.2]	38.5 [35.8,41.2]	38.0 [35.5,40.6]


### Risk factors

Obesity was more common in women (38.6%) than in men (30.7%); this same pattern was observed for central obesity, with 86.9% and 71.7% in women and men, respectively. Among women, 30.4% presented HTA and 17.4% diabetes; among men, it was 32.7% and 14.3%, respectively. For lipids, 67.2% of women and 42.8% of men exhibited low HDL-C levels. Furthermore, we identified that 15.7% of the men had a history of heart attack in their father and/or mother, and of these cases, 80.6% had their first heart attack before 50 years old; among women, 19.5% had this history, and 79.4% had their first heart attack before 50 years old. Finally, a high 10-year risk of fatal cardiovascular disease was observed in 38.1% of men aged 40–79 years, compared with 22.4% of women (Table 2).

**Table 2:** Prevalence of cardiovascular risk factors and the onset of CVD in adults by sex. Mexico, Ensanut 2018.


CHARACTERISTIC	# RISK FACTOR	ALL	MEN	WOMEN

*Sample size in adults 20 years or more*		10,503	4,502	6,001

*Frequency in thousand*		82,767	37,551	45,216

***CVD RISK FACTORS***				

***Overweight or obesity***				

*Overweight (25 ≥ BMI < 30 kg/m*²*)*		39.6 [38.0,41.3]	42.0 [39.6,44.5]	37.7 [35.4,40.0]

*Obesity (BMI ≥ 30 kg/m²)*	***F1***	35.0 [33.4,36.6]	30.7 [28.3,33.1]	38.6 [36.5,40.7]

*Central obesity IDF (WC ≥ 90 in men and ≥ 80 cm in women)*		80.0 [78.7,81.3]	71.7 [69.4,73.9]	86.9 [85.3,88.4]

***Hypertension (HTA)***				

*Previous medical diagnosis of HTA*		18.5 [17.2,19.8]	15.7 [14.0,17.5]	20.8 [19.1,22.6]

*Hypertension treatment*		68.4 [65.0,71.5]	61.3 [55.5,66.9]	72.8 [68.9,76.3]

*Undiagnosed hypertension (140/90 mm Hg)*		13.0 [11.8,14.2]	17.0 [15.3,19.0]	9.6 [8.4,11.1]

*Hypertension + (Dx or Tx or 140/90 mm Hg)*	***F2***	31.5 [29.9,33.1]	32.7 [30.5,35.1]	30.4 [28.4,32.5]

***Diabetes (T2DM)***				

*Previous medical diagnosis of T2DM*		10.3 [9.4,11.3]	9.1 [7.9,10.4]	11.4 [10.1,12.8]

*T2DM treatment*		85.7 [82.4,88.5]	82.6 [77.3,87.0]	87.8 [83.2,91.2]

*Undiagnosed diabetes (Glu ≥126 or HbA1c ≥ 6.5)*		5.7 [5.0,6.5]	5.2 [4.2,6.4]	6.1 [5.1,7.2]

*Insulin Resistance (METS-IR index ≥ 51.13)*		55.0 [48.1,61.8]	46.6 [36.8,56.7]	61.0 [51.7,69.5]

*Total Diabetes (Dx or Glu ≥ 126 or HbA1c ≥ 6.5)*	***F3***	16.0 [14.9,17.2]	14.3 [12.7,16.0]	17.4 [15.8,19.1]

***Lipides, mg/dL***				

*Hypercholesterolemia (TC ≥ 200)*		33.8 [32.1,35.6]	33.8 [31.4,36.4]	33.8 [31.7,36.1]

*High LDL-C (LDL-C ≥100)*		57.6 [55.6,59.6]	57.0 [54.0,60.0]	58.0 [55.5,60.5]

*Low HDL-C (< 40 in men and < 50 in women)*		56.1 [54.4,57.8]	42.8 [40.3,45.3]	67.2 [65.0,69.4]

*Hypertriglyceridemia (TGs ≥ 150)*		56.9 [55.2,58.6]	62.8 [60.4,65.1]	52.1 [49.8,54.4]

*Dyslipidemia (TC ≥ 200 or LDL-C ≥ 100 or Low HDL-C)*	***F4***	84.6 [83.3,85.8]	79.9 [77.7,81.8]	88.5 [86.9,90.0]

*Remnant cholesterol ≥ 30 mg/dL*		46.3 [44.4,48.3]	49.1 [46.1,52.1]	44.4 [41.9,46.9]

***Family history (father and/or mother)***				

*Hypertension*		51.6 [49.9,53.3]	49.6 [46.9,52.3]	53.2 [51.0,55.5]

*Heart attack (HA)*	***F5***	17.7 [16.4,19.1]	15.7 [13.9,17.6]	19.5 [17.7,21.4]

*Before 50 years had his/her first HA*		79.8 [74.9,84.0]	80.6 [72.7,86.7]	79.4 [72.7,84.7]

***Modifiable lifestyle***				

*Physical activity*				

*Vigorous and moderate*		79.7 [78.3,81.0]	83.2 [81.1,85.0]	76.8 [74.8,78.6]

*Inactive*		20.3 [19.0,21.7]	16.8 [15.0,18.9]	23.2 [21.4,25.2]

*Current smoker*	***F6***	18.6 [17.2,20.0]	29.9 [27.5,32.3]	9.2 [7.9,10.6]

*Excessive drinking^a^*		58.5 [55.6,61.4]	67.2 [63.9,70.4]	43.3 [38.9,47.7]

***Others***				

*Low-eGFR, eGFR < 60 ml/min/1.73 m*		2.2 [1.8,2.6]	2.5 [1.9,3.3]	1.9 [1.4,2.5]

*High CVD risk (CVD-risk estimated by Globorisk equation ≥ 10%)^b^*		29.4 [27.4,31.5]	38.1 [34.9,41.4]	22.4 [20.3,24.7]

***ONSET OF CVD***				

***At least one diagnosed CVD event***		5.7[4.7,6.9]	5.7[4.2,7.8]	5.6[4.4,7.2]

*Heart attack*		2.6[2.0,3.2]	2.7[2.0,3.7]	2.4[1.7,3.4]

*Angina pectoris*		2.5[1.8,3.5]	3.0[1.7,5.2]	2.2[1.6,3.1]

*Heart failure*		2.4[1.8,3.2]	1.9[1.3,2.8]	2.7[1.8,4.2]


^a^This pattern corresponds to consuming five or more drinks (male), or four or more drinks (female), the last 30 days. ^b^10-year risk estimates for individuals 40–79 years of age.

**Figure 1:**
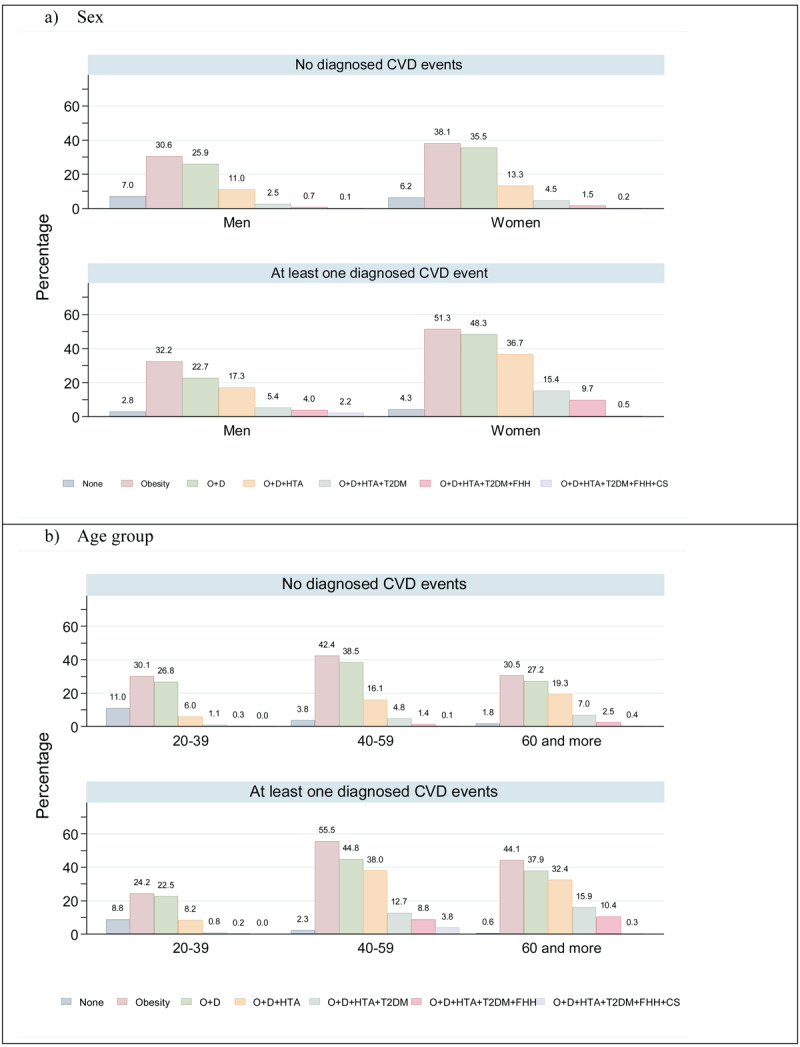
Risk factors associated with CVD in the adult population acording to CVD diagnosis and sex and age. Mexico, Ensanut 2018. Definitions: O: Obesity, D: Dyslipidemia, HTA: Hipertensión, T2DM: Diabetes, FHH: Family history of heart attack and CS: Current smoker. Sample size in adults 20 years or more:12,796 and frecuency in thousand 96,476.

Women exhibited more risk factors than men, independent of CVD diagnosis. Among women, the risk factors were more prevalent in those with CVD diagnoses. The most highlighted factors were obesity (51.3%) and obesity with dyslipidemia (48.3%). Regardless of diagnosis, across all age groups, the most prevalent risk factors in adults aged 40–59 were obesity and the presence of both obesity and dyslipidemia (Figure 1a and 1b).

We found that a high percentage of adults aged 20 or older with at least one CVD diagnosis and medical treatment (statins and antihypertensives) did not meet therapeutic goals, particularly those related to waist circumference (91.2%), body weight (87.4%), and lipid control (85.4%) (Table 3).

**Table 3:** Adult characteristics according to pharmacological treatment. Mexico, Ensanut 2018.


THERAPEUTIC TARGET	AT LEAST ONE DIAGNOSED CVD			

	ALL	TREATMENT USE^a^		

		WITH TREATMENT	WITHOUT TREATMENT	NOT RESPONDED

*Sample size in adults 20 years or more*	415	255	50	110

*Frequency in thousand*	2,947	1,692	452	803

***Demographics***				

Sex, men	49.8 [42.0,57.6]	49.6 [40.0,59.3]	64.5 [45.9,79.6]	41.9 [27.4,57.9]

Age, years (mean)				

20–39	29.8 [23.0,37.6]	15.9 [8.8,27.0]	32.6 [16.7,53.8]	57.5 [41.1,72.3]

40–59	32.7 [26.0,40.2]	34.1 [25.4,43.9]	42.5 [24.5,62.6]	24.3 [13.2,40.4]

60 and more	37.5 [30.6,44.9]	50.0 [40.4,59.6]	24.9 [12.4,43.8]	18.2 [8.4,35.1]

***Family history (father and/or mother)***				

Hypertension	64.6 [56.4,72.0]	64.8 [54.7,73.8]	47.3 [27.7,67.6]	73.6 [58.1,84.9]

Heart attack (HA)	34.8 [27.6,42.7]	40.9 [31.6,50.9]	18.5 [7.7,38.3]	30.9 [17.6,48.4]

Before 50 years had his first HA	67.8 [48.1,82.7]	65.6 [40.8,84.0]	100.0	71.2 [39.2,90.4]

***Body composition***				

Normal weight (18.5 ≥ BMI < 24.9 kg/m²)	16.1 [11.6,21.9]	12.6 [8.0,19.3]	23.3 [11.2,42.2]	19.6 [10.1,34.6]

Noncentral obesity (WC < 90 in men and < 80 cm in women)	10.5 [84.9,15.1]	8.8 [5.2,14.6]	8.6 [3.9,17.9]	15.3 [7.7,28.0]

Lost five kilos or more of weight in the last year	21.5 [15.3,29.2]	23.4 [15.9,33.1]	24.7 [10.6,47.5]	15.6 [6.9,31.6]

***Control***				

Hypertension Arterial (systolic < 140 mmHg and diastolic < 90 mmHg)	71.6 [22.2,77.8]	68.8 [59.9,76.5]	56.6 [36.0,75.2]	86.0 [71.1,93.9]

Diabetes (80 ≥ Glu ≤ 130 mg/dL and HbA1c ≤ 7%)	73.9 [66.6,80.1]	74.1 [65.1,81.5]	80.7 [61.6,91.6]	69.6 [53.0,82.3]

Lipid control (LDL-C < 100 or HDL-C ≥ 40 in men and ≥ 50 in women)	18.8 [74.1,25.9]	14.6 [8.5,24.0]	19.7 [9.7,35.9]	27.2 [15.2,43.7]

***Lifestyle***				

Ex-smoker or never smoker	80.3 [13.7,86.3]	86.1 [75.8,92.5]	88.7 [70.8,96.2]	63.4 [46.8,77.3]

Physical activity (moderate and vigorous: at least 150 minutes per week)	77.6 [16.4,83.6]	70.9 [60.0,79.8]	87.5 [69.1,95.6]	85.2 [73.6,92.3]


^a^Treatment use included stains and hypertensive agents.

### Association of cardiovascular factors with CVD diagnosis

When only one risk factor was considered, we found different results across the treatment use strata (with and without treatment) and sex. Without treatment use, among the men, a significant increase in the risk associated with hypertension [aOR = 2.3; 95% CI: 1.3,4.1] and diabetes [aOR = 2.6; 95% CI: 1.4,4.9] was observed; among the women, both hypertension [aOR = 3.2; 95% CI: 2.1,4.8] and a family history of heart attacks [aOR = 2.2; 95% CI: 1.4,3.4] were found to be significant risk factors. When considering the use of treatment, a notable risk was evident among women with hypertension, diabetes, and a familial predisposition to heart attack; hypertension elevates the likelihood [aOR = 2.4; 95% CI: 1.2, 4.8] of experiencing a cardiovascular event, while a family history exacerbates the risk of CVD [aOR=1.8; 95% CI: 1.0, 3.1]; conversely, diabetes appeared to confer a protective effect [aOR = 0.5; 95% CI: 0.3, 0.9] (Table 4).

**Table 4:** Adjusted risk of having cardiovascular disease^a^ in the presence of a major risk factor for CVD in México, Ensanut 2018.


MODEL	RISK FACTOR	ADJUSTING FOR TREATMENT USE^b^				WITHOUT ADJUSTING FOR TREATMENT USE			
	
		MEN		WOMEN		MEN		WOMEN	
			
		aOR [95% CI]	p VALUE	aOR [95% CI]	p VALUE	aOR [95% CI]	p VALUE	aOR [95% CI]	p VALUE

	*Sample size in adults 20 years or more*	3,879		5,248		3,915		5,303	

	*Frequency in thousand*	33,361		40,506		33,623		40,927	

M1x	Obese	0.7[0.4, 1.4]	0.32	1.6[0.9, 2.8]	0.09	0.8[0.5, 1.4]	0.47	1.3[0.8, 2.1]	0.22

M1x	Dyslipidemia (DLD)	1.7[0.8, 3.7]	0.16	1.4[0.6, 2.9]	0.42	1.7[0.9, 3.1]	0.10	1.1[0.7, 1.9]	0.66

M1x	Hypertension (HTA)	1.6[0.8, 3.4]	0.21	2.4[1.2, 4.8]	0.01	2.3[1.3, 4.1]	0.005	3.2[2.1, 4.8]	< 0.001

M1x	Diabetes (T2DM)	1.9[1, 3.7]	0.06	0.5[0.3, 0.9]	0.02	2.6[1.4, 4.9]	0.003	0.7[0.4, 1.2]	0.21

M1x	First-order family history of heart attack (FHH)	1.1[0.6, 2.3]	0.74	1.8[1, 3.1]	0.04	1.5[0.8, 2.8]	0.19	2.2[1.4, 3.4]	0.001

M1x	Current smoker (CS)	0.9[0.4, 2]	0.82	0.7[0.3,1.9]	0.50	1.2[0.6, 2.1]	0.61	1.8[0.8, 4.4]	0.19

M1x	Obese	0.8[0.4, 1.6]	0.55	1.9[1.1, 3.2]	0.02	1.1[0.6, 1.9]	0.76	1.7[1.1, 2.7]	0.02

M2x	Obese + DLD	0.6[0.3, 1.2]	0.14	1.9[1.1, 3.2]	0.02	0.8[0.5, 1.5]	0.55	1.7[1.1, 2.6]	0.03

M3x	Obese + DLD + HTA	0.8[0.4, 1.7]	0.57	2.6[1.5, 4.5]	0.001	1.6[0.9, 3]	0.13	3.4[2.2, 5.3]	< 0.001

M4x	Obese + DLD +HTA+T2DM	1.1[0.4, 3.4]	0.84	1.2[0.6, 2.4]	0.68	2.2[0.8, 6.3]	0.12	2.5[1.2, 5.1]	0.01

M5x	Obese + DLD +HTA+T2DM+FHH	2.6[0.5, 12.0]	0.24	1.6[0.7, 3.9]	0.30	6.1[1.5, 24.2]	0.01	4.5[1.7, 12]	0.002

M6x	Obese + DLD +HTA+T2DM+FHH+CS	22.4[4.2, 120.5]	< 0.001	1.7[0.2, 17.8]	0.67	24.3[3.2, 186.4]	0.002	2.8[0.3, 26.9]	0.38


aOR: adjusted odds ratio. All models were adjusted for age, physical activity, total energy, and socioeconomic status; additionally, the women’s model included postmenopausal status. ^a^Cardiovascular diseases include heart attack, angina pectoris, and heart failure. ^b^Treatment use included statins and anti-hypertensive agents.

When considering the addition of risk factors and analyzing them together according to treatment use, we observed a significant risk of CVD-associated obesity and dyslipidemia together in untreated women [aOR = 1.7; 95% CI: 1.1, 2.6]; similarly, a risk for obesity, dyslipidemia, and hypertension [aOR = 3.4; 95% CI: 2.2, 5.3], and obesity, dyslipidemia, hypertension, and diabetes [aOR = 2.5; 95% CI: 1.2, 5.1] was found. The same patterns were observed across these combinations, regardless of sex, in models for adults with treatment use, except for the combinations that included diabetes (Table 4).

## Discussion

This study, conducted with a representative sample of Mexican adults, reveals that six out of every 100 adults had experienced a cardiovascular event (heart attack, angina pectoris, or heart failure). Furthermore, it disclosed a significant prevalence of various principal CVD risk factors and disparities between men and women; the predominant risk factors in the female cohort were dyslipidemias (88.5%), obesity (38.6%), familial history of heart attack (19.5%), and diabetes (17.4%), whereas in the male group, the most prevalent risk factor was current smoking (29.9%). This could be explained by the pathophysiology of dyslipidemia, which varies between men and women due to the hormonal alterations women undergo during and post-menopause, resulting in adverse modifications in their lipid profiles (18); also, it is noteworthy that 57% of the women were aged 40 or older. Our data indicated that women exhibited a higher propensity for obesity (as measured by BMI and WC) compared to men; our results are in line with other cohort studies conducted in Asia, Europe, America, and China, where elevated adiposity serves as an independent risk factor for CVDs in women (19,20,21,22,23,24,25). Numerous studies suggest that women are more predisposed than males to diabetes, a significant risk factor for stroke (19,20,26). Moreover, women with diabetes have been shown to have a higher cardiovascular risk, particularly for obstructive coronary artery disease, compared with men (27). Regarding type 2 diabetes, the prevalence in Mexico, according to the ENSANUT, has been higher in women than in men since 2006 (28,29,30,31). Our findings are in line with these reports: 17.4% in women and 14.3% in men. Likewise, previous studies have shown that women with diabetes have a higher cardiovascular risk than men (32,33). On the contrary, we found that diabetes was a protective factor against cardiovascular events; however, after adjusting for concomitant comorbidities such as obesity, dyslipidemia, and hypertension, diabetes became a significant risk factor. This may be explained by the chronic and progressive nature of CVD, which frequently develops in individuals with long-standing metabolic disorders and multiple comorbid conditions (24). Regarding the family history of a heart attack, it was another important risk factor for women; 19.5% of them had antecedents, compared to 15.7% of men; this differs from the findings of Richie Nansseu et al. (26). Hypertension is widely recognized to significantly elevate the risk of cardiovascular events in both the general population and women (27). We found that men had a slightly higher prevalence of hypertension than women (32.7%), although this difference was not statistically significant. Additionally, findings from other studies have corroborated our results (19,34,35). Moreover, research has shown that women’s increased adiposity intensifies the traditional risk factors of diabetes, dyslipidemia, and hypertension (36).

However, we observed that more women than men have two or more risk factors concurrently in those adults who had a heart attack, angina, or heart failure. This may be because women are more likely to have cardiovascular events due to certain health issues they encounter throughout their lives. Preeclampsia, diabetes during pregnancy, hormonal and inflammatory changes after menopause are among these disorders that might affect total cholesterol, No-HDL-cholesterol, LDL-cholesterol, and lipoprotein (a) levels (36). In terms of age groups, people aged 40–59 are more likely to have two or more risk factors. Such conditions can result from gradual changes in lifestyle choices (37).

According to our results, 91% of women with the six risk factors for CVD—obesity, dyslipidemia, hypertension, diabetes, family history, and smoking—have not received a diagnosis of a CVD event, compared to 47% of men. Thus, individuals who do not receive proper pharmacologic treatment and follow-up may be more likely to present a cardiovascular event and/or complications related to it.

It is important to note that there have been various initiatives in Mexico aimed at reducing cardiovascular risk factors, such as the Specific Action Program for the Prevention and Control of Obesity and Cardiovascular Risk (2013–2018) (38) and the HEART program (2021) (39). However, our results indicate that the prevalence of the major risk factors remains high, such as obesity, dyslipidemia, and current smoking, which are associated with behavioral patterns that can be modified with a lifestyle change: a healthy diet and physical activity. These changes not only help manage a healthy weight and improve lipid profiles but also significantly reduce the presence of chronic diseases.

Using statins is emphasized as an important part of secondary prevention of cardiovascular events in adults (40). However, according to our results, persons using statins and some antihypertensives exceed the prescribed thresholds for waist circumference (91.2%), body weight (87.4%), and dyslipidemia (85.4%). These findings could indicate that the pharmaceutical intervention alone is insufficient to achieve the therapeutic goals. Research from several studies indicates that overweight, obesity, and dyslipidemia are associated with prolonged unhealthy lifestyles (41), which may ultimately result in metabolic disorders and subsequent problems or comorbidities leading to cardiovascular events or fatal outcomes (42,43).

We observed in the women’s group that a previous diagnosis of a cardiovascular event was significantly associated with the presence of obesity and dyslipidemia simultaneously, as well as with these two conditions and hypertension, regardless of whether they were using pharmacological treatment. A significant factor that stands out in women is also a family history of heart attacks. In the case of men, a significant risk is observed only for hypertension and diabetes when independent risks are considered in the model and treatment use is not adjusted for. The scientific literature has documented that women are at greater risk of experiencing a cardiovascular event than men (44,45,46,47). This could be explained by the physiological processes they face throughout their lives, which make them more vulnerable. During pregnancy, women are susceptible to gestational diabetes and preeclampsia. After pregnancy, weight gain is common in women, and after menopause, because of hormonal changes, visceral fat accumulation is more likely, and finally, adverse effects on lipids (48). After menopause, the decline in estrogen levels promotes visceral fat accumulation, alters lipid metabolism, raising triglycerides and LDL while lowering HDL, and reduces fatty acid oxidation, increasing the risk of insulin resistance and CVD (49,50).

In general, after controlling medication use, we found that risk factors did not significantly decrease in either the men’s or women’s group. These findings could be related to poor treatment adherence and unhealthy lifestyles maintained over many years, such as an unhealthy diet and a sedentary lifestyle. These factors, which are critical for maintaining metabolic control, have been proposed in health guidelines—for example, those of the American Heart Association (AHA) and the American College of Cardiology (ACC) (51).

According to a study conducted in the United States, women have more risk factors than men, and the Hispanic/Latino population is more likely to have a second cardiovascular event (52). The women also showed unhealthy cholesterol levels, which were later connected to their lack of social security and ultimately resulted in a decrease in the use of statins. As stated by the same study, a large number of participants who had previously experienced a stroke were aware of their vascular risk factors; yet, the data suggest that their treatment was insufficient.

In our study, one limitation we acknowledge is that the cross-sectional design of the survey does not allow us to collect longitudinal information on the duration of lifestyles or on barriers to treatment adherence. The physical activity measure is constrained by its reliance on an index that quantifies the duration of an individual’s movement between locations, potentially leading to overestimation of activity levels. Another limitation is that some of the data are based on participants’ answers, which could introduce bias; however, participants were asked by trained personnel. The final, but equally important limitation is that the ENSANUT survey doesn’t have sufficient information to evaluate women-specific cardiovascular risk factors, such as polycystic ovary syndrome, preeclampsia, menopause, systemic arterial hypertension during pregnancy, rheumatic diseases, hypothyroidism, and gestational diabetes.

One advantage of our study is that the data originates from a nationally representative poll, providing a thorough perspective on the situation across Mexico and on the sex-related differences observed.

Based on our findings, we recommend some actions that could support CVD prevention strategies and the strengthening of Mexican primary health care from a gender perspective: 1) implement targeted screening in populations at higher risk of CVD, incorporating a sex-specific approach. In women, screening should prioritize the detection of abdominal obesity, dyslipidemia, and diabetes mellitus, given their higher prevalence and clustering of cardiometabolic risk factors, while in men, particular emphasis should be placed on the early detection of hypertension and diabetes mellitus. All individuals identified through screening should undergo appropriate diagnostic evaluation; 2) strengthen the medical management of individuals diagnosed with cardiometabolic comorbidities, with an integrated and continuous care approach. In women, this should focus on the simultaneous management of multiple coexisting conditions, whereas in men, priority should be given to optimal control of hypertension, especially when accompanied by diabetes mellitus, and finally; 3) the Mexican health system should focus the prevention actions on the younger adults, in particular to promote healthy lifestyles.

## Conclusion

The prevalence of cardiovascular events was similar between sexes; however, regarding the risk factors, different risk factor profiles were identified, which calls on the Mexican health systems to focus on sex-specific prevention and management strategies. It is necessary to determine why pharmacological treatment alone has not been sufficient to achieve therapeutic goals.
